# Ultrasound-Assisted Coupled with Resin-Based Purification for Sustainable Extraction of Steviosides from *Stevia rebaudiana* Leaves

**DOI:** 10.3390/molecules30163416

**Published:** 2025-08-19

**Authors:** Zidan Liu, Linyu Luo, Zhiqiang Ding, Weihao Long, Tolbert Osire, Qiong Li, Qianfeng Chen, Mengfei Long

**Affiliations:** 1 College of Pharmaceutical Sciences, Southwest University, Chongqing 400715, China; shmily0920@email.swu.edu.cn (Z.L.); lly4566@email.swu.edu.cn (L.L.); spidermande@email.swu.edu.cn (Z.D.); long156836@email.swu.edu.cn (W.L.); liqiong8011@126.com (Q.L.); cqf2011@swu.edu.cn (Q.C.); 2 Faculty of Biology, Shenzhen MSU-BIT University, Shenzhen 518172, China; 6420210017@smbu.edu.cn

**Keywords:** stevioside, extraction, impurity removal, desalting, decolorization

## Abstract

Stevioside, a natural high-intensity sweetener, is widely employed across the food, pharmaceutical, and daily chemical industries due to its intense sweetness and health benefits. However, traditional extraction and purification processes for steviol glycosides from *Stevia rebaudiana* are plagued by low efficiency, high energy consumption, substantial environmental impact, and inconsistent product quality. This study systematically optimized the extraction, decolorization, decontamination, and desalting processes to overcome these challenges. The extraction method was refined using 20% ethanol as the solvent, an optimal temperature of 50 °C, and a 1:10 material-to-liquid ratio, increasing the steviol glycoside yield from 32.0% to 49.1%. Decolorization employing a combination of resins D940 and T5 achieved decolorization rates of 89–92% with minimized steviol glycoside loss, surpassing the non-selective adsorption limitations of activated carbon. For decontamination, calcium hydroxide (Ca(OH)_2_) outperformed diatomaceous earth, attaining a 98% protein removal rate while maintaining steviol glycoside loss below 20%. The desalting resin LXP-016 demonstrated superior performance at 40 °C, enhancing the ability of ionic impurity removal. These optimizations collectively improve the efficiency, sustainability, and quality of steviol glycoside production, offering a promising framework for industrial-scale applications.

## 1. Introduction

*Stevia rebaudiana* Bertoni, commonly known as stevia, is a natural sweetener plant valued for its potent sweetness and negligible caloric content. Its primary sweetening compounds, stevioside and rebaudioside A, are diterpene glycosides with sweetness up to 300 times that of sucrose [1]. These properties position stevia as a compelling alternative to artificial sweeteners, with growing applications in the food and pharmaceutical industries, including beverages, baked goods, and functional foods [2,3].

The extraction of steviosides from *Stevia rebaudiana* leaves is a complex process influenced by factors such as solvent type, extraction method, temperature, and duration, where these parameters are critical to maximizing the yield and purity of these sweeteners. Various techniques have been developed, including conventional methods like maceration, percolation, and solvent extraction, as well as advanced approaches such as ultrasound-assisted extraction (UAE) [4], microwave-assisted extraction (MAE) [5], and pressurized hot water extraction (PHWE) [6]. Among these methods, ultrasound-assisted extraction (UAE) has gained prominence due to its efficiency in reducing extraction time and increasing the yield of bioactive compounds [7,8,9].

Solvent selection is a critical factor in the extraction of steviosides from *Stevia rebaudiana* leaves. Ethanol, methanol, and water are commonly used solvents, with water-based mixtures preferred for their eco-friendliness and non-toxicity [10,11]. However, achieving optimal yield and purity requires precise control of solvent concentration, extraction temperature, and duration. Studies by Ameer et al. [12] and Jentzer et al. [13] have demonstrated that response surface methodology (RSM) and artificial neural network (ANN) modeling effectively predict optimal extraction conditions, enhancing stevioside yield while minimizing solvent consumption.

Despite these advances, significant challenges persist in stevioside extraction, including high operational costs, environmental impact from solvent and energy use, and difficulties in achieving high-purity extracts with minimal loss of active compounds [14]. For instance, conventional methods often require prolonged extraction times, while advanced techniques like MAE and PHWE face scalability issues and high equipment costs [15,16,17]. Moreover, purification processes, such as decolorization and decontamination, frequently result in substantial stevioside loss, compromising product quality and economic viability [18]. Recent efforts to enhance sustainability through novel techniques, such as thin-film microextraction with natural deep eutectic solvents [19], have shown promise in improving yield and environmental impact but are limited by complex implementation and insufficient optimization for industrial scales. To address these limitations, advanced extraction and purification strategies integrating energy efficiency, solvent recovery, and waste reduction are essential for sustainable and cost-effective stevioside production [20,21].

In this study, the objective was to develop an efficient and sustainable method for extracting and purifying steviosides from *Stevia rebaudiana* leaves, addressing challenges in yield, purity, and environmental impact. To achieve this, key extraction parameters, including solvent type, temperature, material-to-liquid ratio, and ultrasonic conditions, were systematically investigated through single-factor experiments. Molecular dynamics simulations were employed to analyze solvent–stevioside interactions, guiding solvent selection. Additionally, decolorization and decontamination processes were refined using selected resins and chemical agents to minimize stevioside loss while improving product purity. Desalination was optimized to further enhance quality. The optimized processes were evaluated for their potential to support scalable, eco-friendly industrial production of high-purity steviosides for food and pharmaceutical applications.

## 2. Results and Discussion

Despite notable progress in steviol glycoside extraction technologies, challenges such as low extraction efficiency, high energy consumption, elevated operational costs, environmental impact, and inconsistent product quality persisted, necessitating further optimization. This study systematically addressed these limitations by optimizing the extraction solvent, temperature, and material-to-liquid ratio, followed by decolorization, decontamination, and desalting, to enhance the yield and purity of steviol glycoside from *Stevia rebaudiana* leaves.

### 2.1. Optimization of Stevia Leaf Extraction

**Figure 1:**
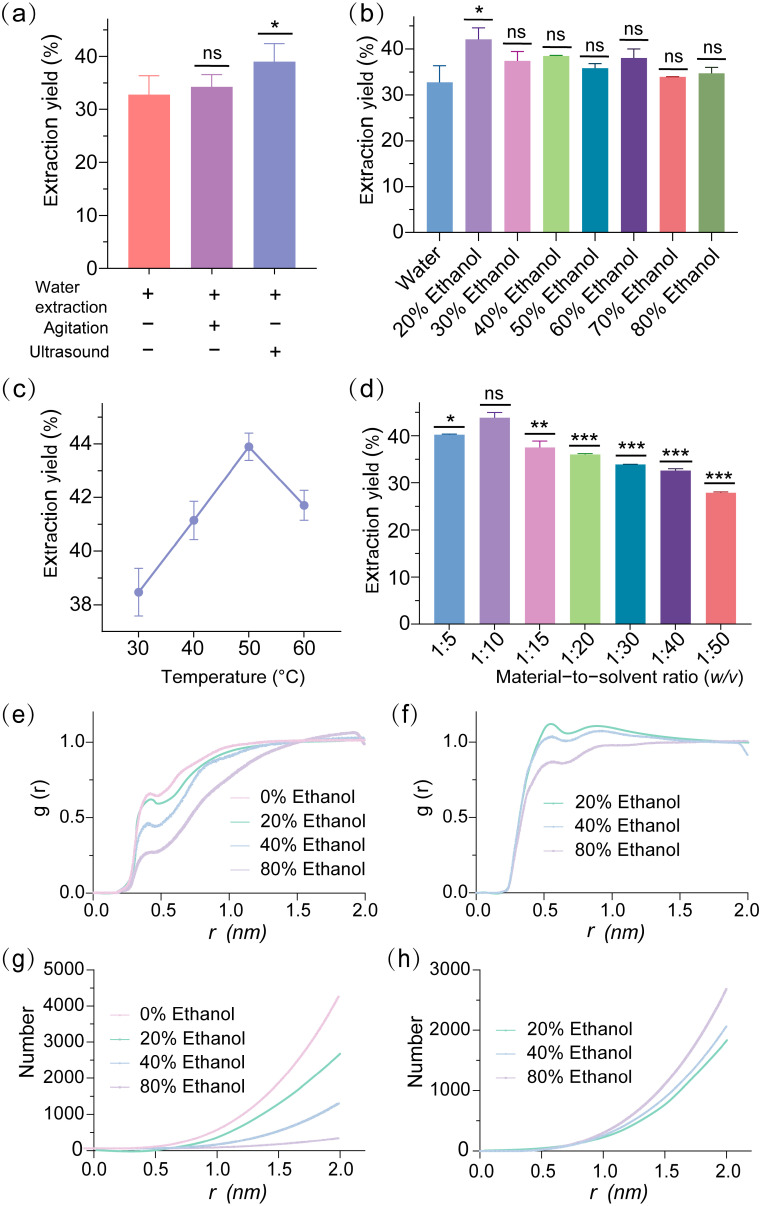
The effects of ultrasonic assistance: (**a**) ethanol concentration(Red: Represents water extraction (+) with no agitation (−) and no ultrasound (−); Purple: Represents water extraction (+) with agitation (+) and no ultrasound (−). Blue: Represents water extraction (+) with no agitation (−) and ultrasound (+)); (**b**) extraction temperature; (**c**) material-to-solvent ratio (*w*/*v*); (**d**) ethanol concentration on water–stevioside RDF g(r) profiles (**e**) ethanol concentration on ethanol–stevioside RDF g(r) profiles; (**f**) ethanol concentration on water–stevioside coordination number; (**g**) and ethanol concentration on ethanol–stevioside coordination number; (**h**) on the extraction yield and molecular interactions of steviol glycosides (ns, not significant, * *p* < 0.05, ** *p* < 0.01, *** *p* < 0.001).

Comparison of water extraction with and without ultrasound (Figure 1a) revealed a modest yield increase from 32.7% to 39.0% with ultrasonic assistance, highlighting its potential to improve efficiency. Effect of ethanol concentration on extraction yield (Figure 1b) identified 20% ethanol as the optimal solvent, achieving the highest yield of 42.1%, significantly outperforming other concentrations (e.g., 50% at 35.8%, 80% at 34.7%), suggesting an optimal balance of solvent polarity and solubility. Optimization of extraction temperature using 20% ethanol with ultrasonic assistance (Figure 1c) peaked at 50 °C with a yield of 43.9%, compared to 38.5% at 30 °C, indicating a temperature-driven enhancement in mass transfer and solubility. Evaluation of material-to-liquid ratio (Figure 1d) established 1:10 as optimal, yielding 43.8% compared to 40.2% at 1:5, underscoring the importance of sufficient solvent volume for effective extraction. Additionally, the ability to remove proteins to some extent with 20% ethanol assisted by ultrasound suggests that the flocculation step can be bypassed in industrial processes, thereby minimizing the loss of steviol glycosides during purification. Comparison of extraction yield before and after optimization confirmed that the optimized process increased the yield in a 200 mL system from 32.0% to 49.1%, a 53.4% improvement, validating the efficacy of the integrated approach. Compared to Wen et al. [4], who reported UAE yields of 35–40% with water, our 49.1% yield with 20% ethanol reflects enhanced solvent–stevioside interactions. Unlike MAE [5], which requires high energy and equipment costs, UAE is more scalable. PHWE [6] achieved similar yields but used higher temperatures, increasing energy consumption. Our method balances efficiency and sustainability.

To further elucidate solvent effects, molecular dynamics (MD) simulations and radial distribution function (RDF) analyses were conducted to investigate the interactions between steviol glycosides and solvents in 0%, 20%, 40%, and 80% ethanol–water mixtures. The RDF profiles for water–steviol glycoside interactions (Figure 1e) revealed that within the first solvation shell (*r* ranged from 0.3 to 0.6 nm), the 0% ethanol condition exhibited the highest g(r) peak value of 0.647, indicating the formation of a robust and densely packed hydrogen bonding network between water molecules and steviol glycosides, reflective of elevated local density under pure water conditions. The 20% ethanol condition displayed a slightly lower peak value of 0.565, characterized by a sharper and narrower peak, suggesting a more concentrated distribution of water molecules and an enhanced hydrogen bonding interaction, which aligns with experimental observations of 20% ethanol as the optimal extraction condition, likely due to its provision of an ideal hydrophobic–hydrophilic balance that facilitates the dissolution and extraction efficiency of steviol glycosides. In contrast, the 40% ethanol condition showed a significantly reduced peak value of 0.452, with a gradual curve ascent, indicating a weakened hydrogen bonding interaction between water and steviol glycosides. The 80% ethanol condition presented a peak value of 0.263, suggesting a progressive weakening of the water–steviol glycoside hydrogen bonding and the introduction of a competitive solvent effect with increasing ethanol concentration, thereby reducing extraction efficiency. The RDF profiles for ethanol–steviol glycoside interactions (Figure 1f) demonstrated that at *r* ranged from 0.3 to 0.5 nm, the 20% ethanol condition exhibited the highest g(r) peak value of 1.135, indicating limited direct interaction between ethanol molecules and steviol glycosides at low concentrations. However, this characteristic may indirectly optimize the extraction process by enhancing the dominant role of water molecules. The 40% ethanol condition displayed a modestly lower peak value of 1.029, with a steeper rise, suggesting an increased involvement of ethanol molecules in the interaction, though this did not translate into improved extraction efficiency. The 80% ethanol condition showed a peak value of 0.871, lower than that of 40%, reflecting a constrained interaction strength but an extended distribution range, potentially attributable to competitive dynamics and spatial repulsion among ethanol molecules at higher concentrations, which likely contributed to a decline in extraction performance.

The coordination number (CN) profiles for water–steviol glycoside interactions (Figure 1g) indicated that the 0% ethanol condition stabilized at a CN of approximately 4000 at r = 2.0 nm, with a smooth ascending trend, suggesting the formation of an extensive solvation layer by water molecules around steviol glycosides in a pure water system. However, this broad distribution may not have achieved optimal extraction efficiency. The 20% ethanol condition exhibited a CN of about 2500, with a modest reduction and a relatively smooth curve, reflecting a moderate decrease in water molecule numbers yet an orderly distribution, a feature consistent with the highest extraction yield observed at 20% ethanol (Figure 1b), highlighting its optimized solvent environment. The 40% ethanol condition declined to a CN of approximately 1000, with a slower rise, indicating a significant reduction in water molecule numbers, while the 80% ethanol condition further decreased to a CN of about 300, with an almost flat curve, underscoring a substantial diminution in water molecule coordination capacity and an unfavorable shift in the solvent environment due to increased ethanol content. CN profiles for ethanol–steviol glycoside interactions (Figure 1h) revealed that the 80% ethanol condition stabilized at a CN of approximately 2500 at r = 2.0 nm, with a rapid initial rise followed by a plateau, suggesting the formation of a robust coordination layer by ethanol molecules at high concentrations, though this did not enhance extraction efficiency. The 40% ethanol condition showed a CN of about 2000, with a slightly slower rise, indicating a moderate coordination capacity at intermediate ethanol levels. The 20% ethanol condition displayed the lowest CN of approximately 1800, with a gradual increase, reflecting a limited coordination role of ethanol molecules at low concentrations; however, this limitation was compensated by the synergistic action of water molecules, achieving optimal extraction efficiency, consistent with the RDF observations in Figure 1f.

### 2.2. Optimization of Decolorization Process

**Figure 2:**
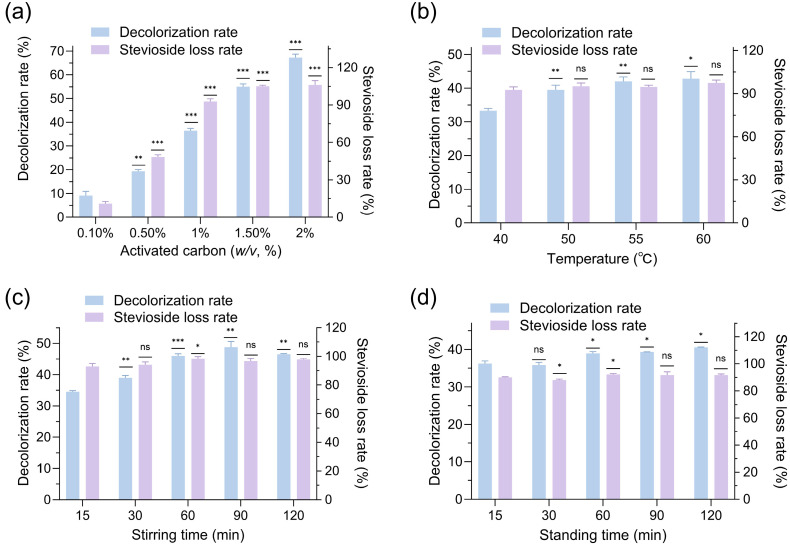
The effects of activated carbon dosage: (**a**) decolorization temperature; (**b**) stirring time; (**c**) and standing time; (**d**) on the decolorization rate and stevioside loss rate of *Stevia rebaudiana* extracts (ns, not significant, * *p* < 0.05, ** *p* < 0.01, *** *p* < 0.001).

Stevioside extracts typically contained a substantial amount of impurities, including chlorophyll, flavonoids, and pigments, which compromised the quality and purity of steviosides, necessitating effective decolorization methods to achieve high-purity steviol glycosides [22,23]. Activated carbon, a porous material known for its large specific surface area and strong adsorption capacity, has been widely utilized in the decolorization of natural plant extracts such as grape seed extract, tea polyphenols, and *Poria cocos* extract [24]. In this study, activated carbon was employed to decolorize stevioside extracts, but the results revealed significant drawbacks. Effect of activated carbon dosage on decolorization and stevioside loss rates (Figure 2a) showed that at 1.5–2.0% activated carbon, the decolorization rate ranged from 55.0% to 67.2%, while steviosides were almost entirely adsorbed, indicating that activated carbon non-selectively adsorbed steviosides alongside pigments. Influence of decolorization temperature on activated carbon performance (Figure 2b) demonstrated that the stevioside loss rate remained consistently high at 92.6–97.4% across temperatures (40–60 °C), suggesting temperature had minimal impact on reducing stevioside adsorption. Effect of stirring time on activated carbon decolorization (Figure 2c) revealed that stevioside loss rates stayed above 93% for stirring durations of 15–120 min, further confirming the strong, non-specific adsorption behavior of activated carbon. Impact of standing time on activated carbon decolorization (Figure 2d) indicated that the decolorization rate reached a maximum value of 40.5% with 30 min of stirring and 120 min of standing at 50 °C, while stevioside loss remained unacceptably high at 91.7%. These findings underscored that activated carbon was unsuitable for stevioside decolorization due to its excessive adsorption of the target compound, prompting the exploration of alternative decolorizing agents.

**Figure 3:**
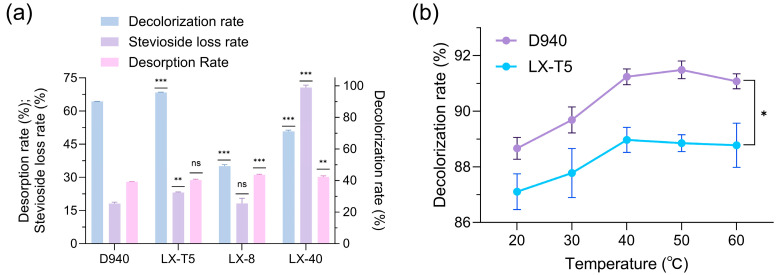
The comparison of decolorization performance among resins D940, LX-T5, LX-8, and LX-40 (**a**) and the influence of decolorization temperature on the performance of resins D940 and LX-T5; (**b**) for Stevia rebaudiana stevioside extracts (ns, not significant, * *p* < 0.05, ** *p* < 0.01, *** *p* < 0.001).

Given the limitations of activated carbon, decolorizing resins were investigated for their potential to preserve stevioside quality without causing physical or chemical damage during the decolorization process. Four decolorizing resins, namely D940, LX-T5, LX-8, and LX-40, were screened for their efficacy. Comparison of decolorization performance of resins D940, LX-T5, LX-8, and LX-40 (Figure 3a) revealed that D940 and LX-T5 exhibited complementary decolorization effects, with LX-T5 achieving a decolorization rate of 95.7%, which was 5.6% higher than D940′s 90.1%, though its stevioside loss rate was also higher at 23.1% compared to D940′s 18.1%. Optimization of decolorization temperature for resins D940 and LX-T5 (Figure 3b) identified 40–50 °C as the optimal range, where decolorization rates improved to 91.3% for D940 while the decolorization rate of LX-T5 was stabilized at 88.8%, which indicated a temperature-dependent enhancement in pigment removal efficiency.

**Figure 4:**
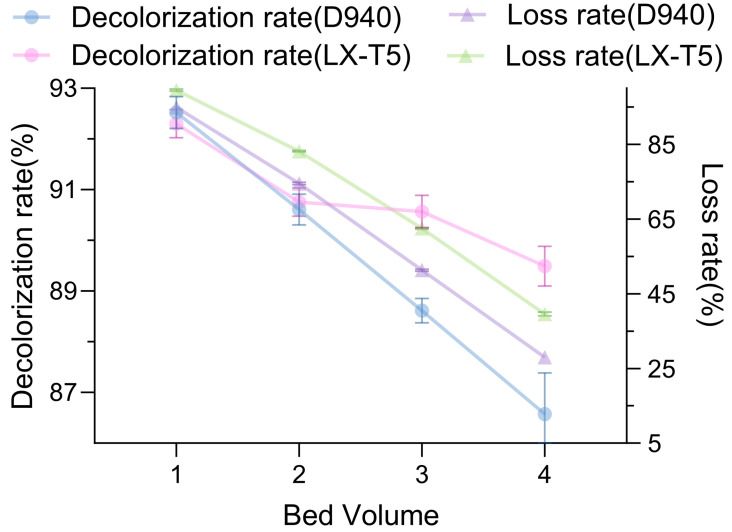
Comparison of decolorization rates of LX-T5 and D940 for large-scale fluid treatment across varying bed volumes.

Comparison of decolorization rates of LX-T5 and D940 for large-scale fluid treatment (Figure 4) demonstrated a widening gap in decolorization rates as fluid volume increased, with LX-T5 achieving a rate of 89.5% compared to D940 achieving a rate of 87.4%, suggesting LX-T5’s superior suitability for large-scale applications. However, LX-T5’s stevioside loss rate of 39% was approximately 12% higher than D940’s 27%, highlighting a trade-off between decolorization efficiency and product retention. In order to balance these factors, we propose a combined approach in which D940 and LX-T5 are proportionally added to the column to take advantage of their complementary roles to achieve high decolorization rates while reducing steviol glycoside loss. Resin-based decolorization, particularly with a proposed hybrid D940/LX-T5 column, could provide a sustainable and efficient alternative to activated carbon, warranting further studies on resin combinations and column configurations.

### 2.3. Comparison of Diatomaceous Earth Decontamination and Ca(OH)_2_ Decontamination

**Figure 5:**
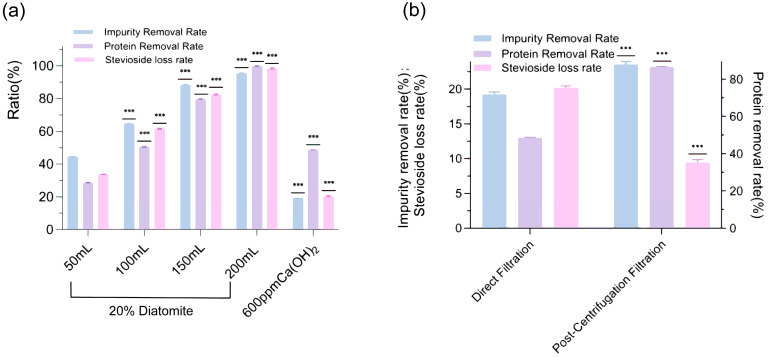
The comparative performance of diatomaceous earth and Ca(OH)_2_ on impurity removal and stevioside retention: (**a**) and the enhancement of decontamination efficiency by Ca(OH)_2_ using direct versus post-centrifugal filtration methods; (**b**) in *Stevia rebaudiana* stevioside extracts (*** *p* < 0.001).

The presence of substantial impurities, such as chlorophyll, flavonoids, and proteins, in *Stevia rebaudiana* leaf extracts posed significant challenges to achieving high-purity steviosides, necessitating effective decontamination strategies to enhance product quality. This study evaluated the efficacy of two de-impurity agents, diatomaceous earth and calcium hydroxide (Ca(OH)_2_), in removing these contaminants while minimizing stevioside loss. Effect of diatomaceous earth and Ca(OH)_2_ dosage on impurity removal and stevioside loss rates (Figure 5a) demonstrated that diatomaceous earth exhibited superior impurity removal, achieving rates of up to 95% at 200 mL slurry volume, but this came at the cost of an excessively high stevioside loss rate, reaching nearly 100% when filtered through a diatom cake, indicating a non-selective adsorption mechanism. In contrast, Ca(OH)_2_ achieved a lower impurity removal rate of 60% at 600 ppm, but its stevioside loss rate remained significantly lower at approximately 20%, with 13% of this loss attributed to filtration through a Büchner funnel, suggesting a more selective purification process. The analysis further revealed that the protein removal rate with Ca(OH)_2_ reached 48% at 600 ppm, compared to 99% with diatomaceous earth at 200 mL, highlighting a trade-off between impurity removal efficiency and stevioside retention. These findings underscore the need for alternative filtration strategies to optimize the decontamination process.

To address the limitations of initial filtration methods, we investigated the impact of different purification techniques on decontamination efficiency. Comparison of direct filtration and post-centrifugal filtration on impurity, protein, and stevioside loss rates (Figure 5b) showed that direct filtration achieved an impurity removal rate of 19%, a protein removal rate of 48%, and a stevioside loss rate of 20%, reflecting moderate purification with notable product loss. In contrast, post-centrifugal filtration significantly enhanced performance, increasing the impurity removal rate to 23%, the protein removal rate to 86%, and reducing the stevioside loss rate by approximately 10%. These results suggested that centrifugation effectively separated precipitated impurities and proteins, thereby minimizing stevioside entrapment during filtration. The enhanced protein removal rate of 86% with post-centrifugal filtration indicated a robust mechanism for targeting proteinaceous impurities, which was critical for improving stevioside purity. Additionally, the reduction in stevioside loss to 10% highlighted the potential of centrifugal filtration to preserve yield, offering a practical advantage over direct filtration methods.

### 2.4. Optimization of Ca(OH)_2_ Decontamination Process

**Figure 6:**
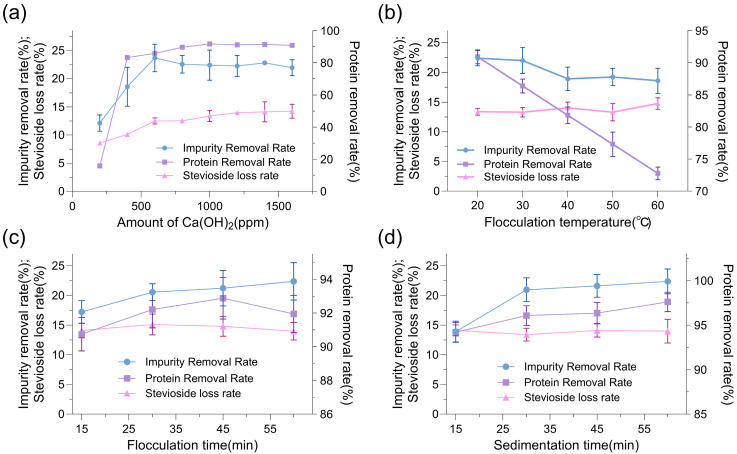
The effects of Ca(OH)_2_ dosage: (**a**) flocculation temperature; (**b**) stirring time; (**c**) resting time; (**d**) on protein removal efficiency and stevioside retention during the decontamination of *Stevia rebaudiana* extracts.

Given the superior performance of Ca(OH)_2_ over diatomaceous earth in balancing impurity removal with minimal stevioside loss, we further optimized the Ca(OH)_2_ decontamination process to maximize protein removal efficiency while preserving stevioside yield. The parameters investigated included Ca(OH)_2_ addition, flocculation temperature, stirring time, and resting time, with the goal of enhancing the overall purity of *Stevia rebaudiana* stevioside extracts. Effect of Ca(OH)_2_ addition on protein removal and stevioside loss rates (Figure 6a) revealed that increasing the Ca(OH)_2_ concentration from 600 ppm to 1000 ppm improved the protein removal rate from 86% to 92%, while the stevioside loss rate remained relatively stable at 12–14%, indicating that 1000 ppm represented an optimal dosage for effective flocculation without compromising product yield. Influence of flocculation temperature on decontamination efficiency (Figure 6b) demonstrated that a temperature of 20 °C yielded the highest protein removal rate of 90.9%, compared to 86.4% at 30 °C and 81.8% at 40 °C, suggesting that lower temperatures enhanced the flocculation of proteinaceous impurities, possibly due to reduced solubility and increased precipitation at 20 °C, while the stevioside loss rate remained consistent within 15% across the temperature range. Optimization of stirring time for Ca(OH)_2_ flocculation (Figure 6c) indicated that stirring for 30–45 min achieved the optimal protein removal rate of 92.8%, with no significant improvement beyond 45 min (e.g., 91.9% at 60 min), while the stevioside loss rate was maintained at 15%, highlighting that a stirring duration of 30–45 min was sufficient for effective mixing and flocculation without causing unnecessary shear stress that could lead to product loss. Impact of resting time on Ca(OH)_2_ decontamination (Figure 6d) showed that a resting period of 30–60 min after stirring resulted in a protein removal rate of 97%, while the stevioside loss rate remained at 14%, confirming that a 30–60 min resting period allowed optimal settling of impurities without affecting stevioside stability. Under these optimized conditions—Ca(OH)_2_ addition of 1000 ppm, flocculation temperature of 20 °C, stirring for 30–45 min, and resting for 30–60 min—the protein removal rate increased from 86% to 97%, representing a 13% improvement in efficiency, while the stevioside loss rate was minimized to 10%. These findings underscored the effectiveness of the optimized Ca(OH)_2_ flocculation process in achieving high-purity stevioside extracts by targeting proteinaceous impurities with minimal impact on the target compound. The consistency of the stevioside loss rate within 15% across all parameters suggested that the optimized conditions not only enhanced decontamination but also ensured product retention, making this process a promising candidate for industrial-scale purification of stevioside extracts.

### 2.5. Optimization of Desalting Process

**Figure 7:**
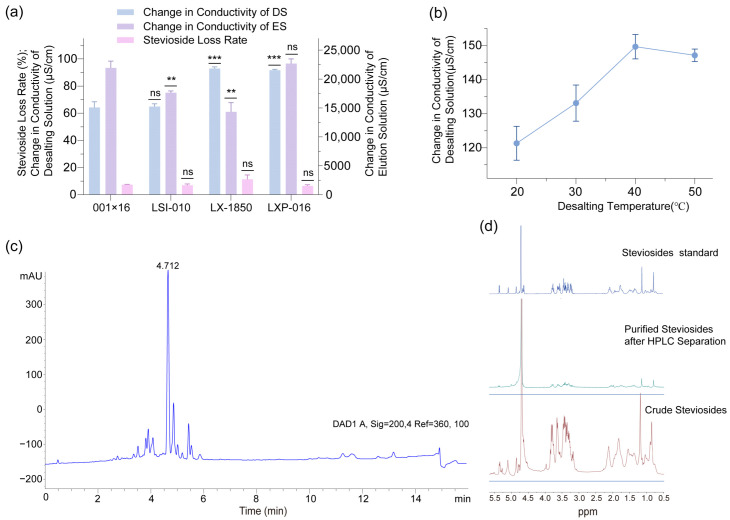
The comparison of conductivity reduction and stevioside retention among different desalting resins including LX-1850 and LXP-016: (**a**) and the effect of desalting temperature on the performance of resin LXP-016; (**b**) during the treatment of *Stevia rebaudiana* stevioside extracts, the HPLC analysis of steviol glycosides extracted from *Stevia rebaudiana* leaves; (**c**) NMR spectra of steviol glycosides; (**d**) (ns, not significant, ** *p* < 0.01, *** *p* < 0.001).

To further elevate the purity and quality of steviosides extracted from *Stevia rebaudiana* leaves, this study employed desalting resins to reduce ionic impurities, a critical step in refining steviol glycoside products for commercial applications. Conductivity measurements, conducted using a conductivity meter, were utilized to assess the changes in conductivity of both the draw solution (DS) and extracted solution (ES) before and after desalting, as well as before and after desorption, to evaluate resin performance and stevioside retention. Comparison of conductivity changes and stevioside loss rates across different desalting resins (Figure 7a) revealed that the change in DS conductivity was comparable between LX-1850 and LXP-016, reaching approximately 91.7 µS/cm. However, the amount of conductivity change of the desorbed solution after LX-1850 treatment was less than that of LXP-016, suggesting a higher efficiency in ionic removal by LXP-016 during desorption. Additionally, the stevioside loss rate with LX-1850 was approximately 11.4%, twice that of LXP-016 at 6.4%, indicating that LXP-016 preserved stevioside yield more effectively while maintaining comparable desalting efficiency. These findings identified LXP-016 as the optimal desalting resin due to its balanced performance in impurity removal and product retention. Effect of desalting temperature on LXP-016 performance (Figure 7b) demonstrated that the change in DS conductivity increased from 121.3 µS/cm at 20 °C to a peak of 149.7 µS/cm at 40 °C, followed by a slight decline to 147.2 µS/cm at 50 °C, reflecting an optimal temperature range for ionic transport and resin efficiency. The stevioside loss rate remained stable at 10% across the temperature range, suggesting that temperature variations had minimal impact on product loss. Consequently, 40 °C emerged as the optimal desalting temperature for LXP-016, as it maximized conductivity change and maintained a low stevioside loss rate. The optimized conditions with LXP-016 at 40 °C not only enhanced the removal of ionic impurities, as evidenced by the peak conductivity change, but also ensured a stevioside retention rate of 90%, representing a significant improvement over the 80% retention observed with LX-1850. This optimization underscored the importance of resin selection and temperature control in the desalting process, offering a robust method to achieve high-purity stevioside extracts. The results also suggested that further investigation into the scalability of the LXP-016 resin system, including the evaluation of continuous flow desalting units and the impact of varying feed concentrations, could enhance its industrial applicability, potentially reducing processing costs and improving consistency in large-scale stevioside production.

The crude extract was subjected to high-performance liquid chromatography (HPLC) for separation. The HPLC results revealed that the characteristic retention time of stevioside was approximately 4.7 min (Figure 7c), which is consistent with the typical retention profile of stevioside. The stevioside collected from HPLC purification was analyzed using nuclear magnetic resonance (NMR) and compared with standard and crude samples (Figure 7d). The purified stevioside exhibited characteristic chemical shifts in the NMR spectrum that aligned with those of the standard, typically observed in the characteristic regions for glycosides and terpenoids, with sharper and clearer signals, fewer impurity peaks, hydrogen spectral integration ratios consistent with the standard, and coupling patterns similar to those of the standard, indicating the intact stereochemical structure of the sugar and terpenoid rings. In contrast, the crude sample spectrum likely showed additional broad or overlapping signals and abnormal integration due to impurity interference. These features collectively validate the effectiveness of the HPLC purification process and demonstrate the high purity of the purified stevioside.

The optimized extraction, decolorization, decontamination, and desalting processes significantly improved stevioside yield, purity, and sustainability, offering a scalable framework for industrial production.

The extraction phase benefited greatly from the integration of ultrasound-assisted techniques with 20% ethanol, which markedly enhanced stevioside yield to 49.1%, representing a 53.4% improvement over baseline methods. This enhancement likely stemmed from ultrasound’s ability to improve mass transfer and solvent penetration, coupled with the optimal solvent polarity of 20% ethanol, which balanced stevioside solubility and extraction efficiency. Molecular dynamics simulations further supported this approach by illustrating how ethanol concentration influenced solvent–stevioside interactions, with 20% ethanol emerging as a practical choice due to its cost-effectiveness and additional benefit of removing proteins. This dual role of the solvent in extraction and protein removal suggests a potential for process simplification, reducing the need for additional flocculation steps in industrial settings, which could lower both energy consumption and stevioside loss. These findings align with prior research emphasizing ultrasound’s role in enhancing plant matrix extraction [25], and they highlight the importance of solvent selection in achieving sustainable production practices.

Decolorization posed another significant hurdle due to the presence of pigments like chlorophyll and flavonoids in the crude extract. While activated carbon initially seemed promising due to its adsorption capacity, its non-selective nature led to excessive stevioside loss, rendering it impractical. Decolorizing resins D940 and LX-T5 achieved decolorization rates of 90.1% and 95.7%, respectively, with stevioside loss of 18.1–23.1%. A proposed hybrid D940/LX-T5 column could leverage its complementary effects to balance efficiency and retention, warranting further investigation [26].

Decontamination efforts targeting impurities like proteins and flavonoids revealed the limitations of diatomaceous earth, which, despite high impurity removal, caused near-total stevioside loss due to non-selective adsorption. Calcium hydroxide (Ca(OH)_2_), however, provided a more balanced alternative, achieving effective impurity removal while keeping stevioside loss at 15% after optimization. The incorporation of centrifugal filtration further enhanced protein removal efficiency to 98%, suggesting that process integration can significantly improve yield preservation. This approach highlights Ca(OH)_2_’s potential as a selective decontaminant, offering a sustainable option for industrial purification by minimizing product loss and targeting proteinaceous impurities effectively.

The desalting process addressed ionic impurities, critical for ensuring stevioside quality in food and pharmaceutical applications. LXP-016 proved to be the optimal resin, balancing ionic removal with a low stevioside loss rate of 6.4%, particularly at 40 °C, where it achieved a 90% retention rate. This optimization underscores the importance of resin selection and temperature control in achieving high-purity steviosides, providing a reliable method for industrial refinement that aligns with industry standards for sweetener purification [27].

Overall, these optimized processes collectively tackled the primary challenges in stevioside production, improving extraction yield, purity, and retention while reducing environmental impact through streamlined steps and eco-friendly reagents. However, limitations such as the scalability of hybrid resin columns and centrifugal filtration systems remain, necessitating large-scale validation. Subsequent research endeavors should concentrate on continuous processing systems, economic viability, and the sensory characteristics of the final product to ascertain its appropriateness for a range of applications. These advancements lay a strong foundation for sustainable and efficient stevioside production, supporting its broader adoption in the food and pharmaceutical industries.

## 3. Materials and Methods

**Figure 8:**
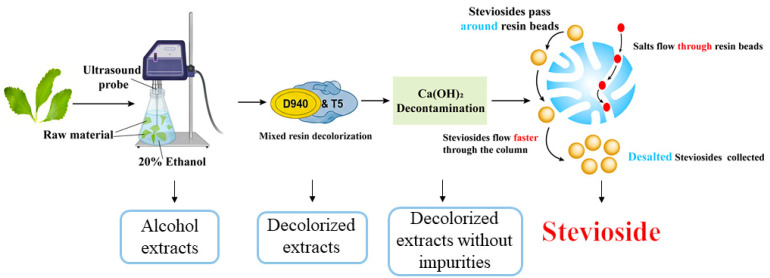
Experimental workflow.

The stevioside production process involved sequential steps starting with ultrasound-assisted extraction (UAE) optimized in Section 3.2. Initial small-scale experiments (0.05 g powder with 1 mL solvent) determined optimal conditions (20% ethanol (*v*/*v*), 50 °C, 1:10 *w*/*v* ratio), scaled up to a 200 mL system, yielding 49.1% stevioside. This extract was used for (i) decolorization with activated carbon (Section 3.3) and resins (Section 3.4), proposing a hybrid D940/LX-T5 approach; (ii) decontamination with diatomaceous earth and Ca(OH)_2_ (Section 3.5) based on the best decolorization result; and (iii) further purification (Section 3.6 and Section 3.7). Each step built on the optimal outcome of the previous one, ensuring a continuous process to enhance yield and purity (Figure 8).

### 3.1. Materials and Reagents

*Stevia rebaudiana* leaves were sourced from the Stevia planting area in Jiuquan, Gansu Province, China, with a yield of 300–350 kg per 700 m^2^ and quality controlled per total glycoside content standards of 12–15%. Stevioside standard was purchased from Chroma Dex, Longmont, CO, USA (content 98.53%). Ethanol (analytical grade) was purchased from Shanghai Aladdin Biochemical Technology Co., Ltd. (Shanghai, China). Activated carbon was acquired from Macklin Biochemical Technology Co., Ltd. (Shanghai, China). Decolorizing resins D940 and T5 were sourced from Anhui Samsung Resin Technology Co., Ltd. (Bengbu, China) and Xi’an Lanxiao Technology Co., Ltd. (Xi’an, China), respectively. Calcium hydroxide (Ca(OH)_2_, analytical grade) was obtained from Sigma Chemical Co. (St Louis, MO, USA). Desalting resins LX-1850 and LXP-016 were purchased from Xi’an Lanxiao Technology Co., Ltd. (Xi’an, China). Diatomaceous earth was acquired from Shanghai Yuanye Biotechnology Co., Ltd. (Shanghai, China). All other chemicals used were of analytical grade or higher.

### 3.2. Optimization of Stevioside Extraction

*Stevia rebaudiana* leaves were ground into a 100-mesh powder using a pulverizer and transferred to a 2 mL Eppendorf tube for extraction. To optimize the extraction method, 0.05 g of powder was mixed with 1 mL of deionized water and subjected to one of three methods: (i) water bath extraction at 30 °C for 30 min, (ii) shaker extraction at 220 rpm and 30 °C for 30 min, or (iii) ultrasonic extraction at 30 °C with 100% power for 30 min. For solvent optimization, 0.05 g powder was extracted with 1 mL of deionized water or ethanol solution (in water) at concentrations (*v*/*v*) of 20%, 30%, 40%, 50%, 60%, 70%, or 80% using ultrasonic extraction at 100% power and 30 °C for 30 min. Based on single-factor experiments showing 20% ethanol yielded 42.1% (Figure 1b), it was selected for temperature optimization, where 0.05 g powder was extracted with 1 mL of 20% ethanol (*v*/*v*) using ultrasonic extraction at 100% power for 30 min at 30 °C, 40 °C, 50 °C, or 60 °C. For material-to-solvent ratio optimization, 0.2, 0.1, 0.05, 0.033, 0.025, or 0.02 g powder was mixed with 1 mL of 20% ethanol (*v*/*v*), corresponding to ratios (*w*/*v*) of 1:5, 1:10, 1:20, 1:30, 1:40, or 1:50, and extracted using ultrasonic extraction at 100% power and 30 °C for 30 min. In all experiments, each extract was filtered, and the residue was subjected to two additional extractions with 1 mL of the respective solvent for 30 min to maximize stevioside recovery. Extraction yield was determined using the anthrone colorimetric method [28]. Briefly, 0.4 mL of sample solution was mixed with 0.1 mL of 1.5% anthrone solution and 1.0 mL of concentrated sulfuric acid, heated in a water bath at 80 °C for 15 min, and the absorbance was measured at 621 nm. A calibration curve was constructed using stevioside standards (0.02–0.6 mg/mL) under identical conditions.

### 3.3. Optimization of Decolorization Process with Activated Carbon

Decolorization used the extracts from Section 3.2 (20% ethanol, UAE, 49.1% yield). To optimize the decolorization of *Stevia rebaudiana* stevioside extracts using activated carbon, the effects of carbon dose, decolorization time, and temperature were investigated in a 1 mL decolorization system. For dose optimization, activated carbon was added at concentrations of 0.1%, 0.5%, 1.0%, 1.5%, or 2.0% (*w*/*v*), and the mixture was stirred at 50 °C and 220 rpm for 30 min, followed by 30 min of standing. To evaluate decolorization time, 1.0% activated carbon was used, and two conditions were tested: (i) stirring at 50 °C and 220 rpm for 15, 30, 60, 90, or 120 min, followed by 30 min of standing, or (ii) stirring at 50 °C and 220 rpm for 30 min, followed by standing for 15, 30, 60, 90, or 120 min. For temperature optimization, 1.0% activated carbon was added, and the mixture was stirred at 40 °C, 50 °C, 55 °C, or 60 °C and 220 rpm for 30 min, followed by 30 min of standing. In all experiments, the mixture was centrifuged at 12,000 rpm for 2 min (twice), and the supernatant was diluted 50-fold. Decolorization and stevioside loss rates were determined by measuring absorbance, with stevioside content quantified using the anthrone colorimetric method as described above.

### 3.4. Selection and Optimization of the Best Decolorizing Resin for Steviol Glycoside Extraction

Resin decolorization used the extracts from Section 3.2 (20% ethanol, UAE, 49.1% yield). To select and optimize the decolorizing resin for *Stevia rebaudiana* stevioside extracts, resin pretreatment, decolorization conditions, and dynamic adsorption were investigated. Resins were pretreated by sequential washing with deionized water, 5% NaOH solution, deionized water, 5% HCl solution, deionized water, 5% NaOH solution, and a final rinse with deionized water. For resin selection, 0.25 g of pretreated resin was mixed with 1 mL of stevioside extract in a 2 mL Eppendorf tube and stirred at 150 rpm and 30 °C for 1 h. To optimize decolorization temperature, the selected resin (0.25 g) was mixed with 1 mL of stevioside extract in a 2 mL Eppendorf tube and stirred at 150 rpm for 1 h at 20 °C, 30 °C, 40 °C, 50 °C, or 60 °C. For dynamic adsorption, 25 g of the selected resin was added to 100 mL of stevioside extract and stirred at room temperature for 1 h. Decolorization rate was determined by measuring the absorbance change at 420 nm before and after decolorization. Desorption rate was assessed by desorbing the resin with 5% NaOH solution for 1 h and measuring absorbance at 420 nm [29].

### 3.5. Investigation of Diatomaceous Earth and Ca(OH)_2_ for Impurity Removal in Steviol Glycoside Extraction

Impurity removal used the extracts from Section 3.4. To evaluate impurity removal in *Stevia rebaudiana* stevioside extracts, diatomaceous earth and calcium hydroxide (Ca(OH)_2_) were investigated. For diatomaceous earth purification, 20% (*w*/*v*) diatomaceous earth slurry was prepared, and portions of 50, 100, 150, or 200 mL were filtered through a Buchner funnel equipped with 12.5 cm slow-speed qualitative filter paper to form diatomaceous cakes of varying thicknesses. A total of 20 mL stevioside extract was passed through each cake to remove impurities. For Ca(OH)_2_ purification, 600 ppm of food-grade Ca(OH)_2_ was added to 20 mL of stevioside extract, and the mixture was stirred at 30 °C and 220 rpm for 30 min, followed by 30 min of standing. The mixture was then filtered using a Buchner funnel with 12.5 cm slow-speed qualitative filter paper, either directly or after centrifugation at 10,000 rpm for 5 min. Impurity removal rate was determined by vacuum freeze-drying, protein removal rate was measured using the Bradford reagent kit [30], and stevioside loss was measured by the anthrone method.

### 3.6. Optimization of Ca(OH)_2_ Purification Process

Ca(OH)_2_ purification used the extracts from Section 3.4. To optimize the Ca(OH)_2_ purification process for *Stevia rebaudiana* stevioside extracts, the effects of Ca(OH)_2_ dosage, flocculation temperature, stirring time, and settling time were investigated in a 20 mL extract system. For dosage optimization, food-grade Ca(OH)_2_ was added at concentrations of 200, 400, 600, 800, 1000, 1200, 1400, or 1600 ppm, with the mixture stirred at 220 rpm and 30 °C for 30 min, followed by 30 min of standing. To evaluate flocculation temperature, a fixed Ca(OH)_2_ dosage of 1000 ppm was used, and the mixture was stirred at 220 rpm for 30 min at 20 °C, 30 °C, 40 °C, 50 °C, or 60 °C, followed by 30 min of standing. For stirring time optimization, the mixture with 1000 ppm Ca(OH)_2_ was stirred at 220 rpm and 30 °C for 15, 30, 45, or 60 min, followed by 30 min of standing. For settling time optimization, the mixture with 1000 ppm Ca(OH)_2_ was stirred at 220 rpm and 30 °C for 30 min, followed by standing for 15, 30, 45, or 60 min. Impurity removal rate was determined by vacuum freeze-drying, protein removal rate was measured using the Bradford reagent kit, and stevioside loss rate was quantified.

### 3.7. Selection and Optimization of Desalting Resin for Steviol Glycoside Extraction

Desalting used the extract from Section 3.6. Post-decolorization and decontamination, resins (LX-1850, LXP-016) were pretreated by washing with water, followed by 5% HCl solution, water, 5% NaOH solution, water, 5% HCl solution, and water. To select the best desalting resin, a system consisting of 0.25 g of resin and 1 mL of steviol glycoside extract in a 2 mL EP tube was tested. The desalting process was performed at 30 °C with stirring at 150 rpm for 1 h. After selecting the optimal resin, the desalting temperature was optimized by testing various temperatures (20 °C, 30 °C, 40 °C, and 50 °C), with 0.25 g of resin and 1 mL of steviol glycoside extract, stirred at 150 rpm for 1 h. Finally, the conductivity was measured before and after desalting, as well as before and after desorption, using a conductivity meter.

### 3.8. Molecular Dynamics Simulation and RDF Analysis

To investigate the molecular interactions of stevioside in ethanol–water mixtures, molecular dynamics (MD) simulations and radial distribution function (RDF) analyses were performed using GROMACS 2023.5 [31]. The stevioside molecule, provided as a mol2 file, was processed with ACPYPE 2023.11.14 [32] to generate topology and coordinate files compatible with the AMBER99SB-ILDN force field [33]. Three systems were constructed with ethanol concentrations of 0% (pure water), 40%, and 80% (*v*/*v*). Each system was assembled in a dodecahedral simulation box with a 1.0 nm edge distance using Packmol 21.0.04 [34]. Ethanol molecules were added to achieve the desired concentrations, and the systems were solvated with TIP3P water [35]. The topology file was updated to incorporate ethanol definitions and counts. System neutrality was ensured by adding Na^+^ and Cl^−^ ions, selecting the solvent group for ion placement. Energy minimization was conducted using the steepest descent algorithm to eliminate steric clashes. Equilibration proceeded in two stages: an NVT ensemble (constant volume and temperature) for 100 ps at 300 K, followed by an NPT ensemble (constant pressure and temperature) for 100 ps at 300 K and 1 bar. Production MD simulations were run for 10 ns to capture solvent–stevioside dynamics. Trajectories were adjusted for periodic boundary conditions and centered on the stevioside molecule using MDAnalysis 2.9.0. RDF analyses quantified solvent interactions by computing the radial distribution function g(r) and coordination number *CN*(*r*) for stevioside with oxygen atoms of water and ethanol, selected based on the stabilization observed in coordination numbers.

### 3.9. Structural Analysis

The structural analysis of compounds in *Stevia rebaudiana* leaves primarily relied on high-performance liquid chromatography (HPLC) and nuclear magnetic resonance (NMR) spectroscopy, including both the total extract and the purified steviosides isolated via HPLC (Shimadzu, Osaka, Japan) for validation. The mobile phase consisted of methanol and acetone in an 80:20 (*v*/*v*) ratio, with a detection wavelength of 210 nm, a flow rate of 0.5 mL/min using a C18 column. Standard Stevioside was prepared individually by dissolving 1 mg in 1 mL of 98% methanol to achieve concentrations ranging from 0.1 to 0.3 mg/mL. Additionally, NMR data were acquired using a Bruker Ascend 400 spectrometer equipped (manufactured by Bruker Corporation, sourced from Rheinstetten, Karlsruhe, Germany) with a cryo-cooled probe, operating at a frequency of 400 MHz for 1H analysis.

### 3.10. Statistical Analysis

Given the small-scale extraction experiments using milligram quantities of raw material and 1 mL of solvent, statistical analysis was conducted to evaluate differences in parameters (e.g., extraction yield, decolorization rate, impurity removal, and stevioside loss) across extracts. Two-sample independent *t*-tests were performed, with each concentration group including three replicate measurements. Welch’s *t*-test was applied to account for potential unequal variances, using a significance level of α = 0.05 (two-tailed). The null hypothesis assumed no difference in mean decolorization rates between paired groups, while the alternative hypothesis posited a significant difference. *p*-values were calculated to assess statistical significance, and a Bonferroni correction (α’ = 0.05/10 = 0.005) was applied to adjust for multiple pairwise comparisons. Analyses were conducted using Python’s SciPy library (version 1.9.3).

## 4. Conclusions

This study successfully optimized the extraction and purification of steviosides from *Stevia rebaudiana* leaves, addressing challenges of low efficiency, high energy use, and environmental impact. Ultrasound-assisted extraction with 20% ethanol increased the yield to 49.1%, a 53.4% improvement, while also facilitating protein removal. The decolorization rate of LX-T5 was comparable to that of D940, and T5 was more suitable for treating a large amount of decolorized solution. However, its steviol glycoside loss rate reached 39%, which was about 12% higher than that of D940. Therefore, it may be possible to proportionally load the two columns to complement each other for better decolorization using a hybrid D940/LX-T5 resin column. Ca(OH)_2_ decontamination, optimized at 1000 ppm and 20 °C, enhanced protein removal to 98% with minimal loss of steviosides. Desalting using LXP-016 at 40 °C ensured a 90% retention rate. Thus, the optimal scheme integrates (1) UAE with 20% ethanol (50 °C, 1:10, 3 × 30 min, ~1.5 h); (2) D940/LX-T5 decolorization (1 h); (3) Ca(OH)_2_ decontamination with centrifugal filtration (1.5–2 h); and (4) LXP-016 desalting (1 h). Total process time was ~3–4 h, yielding 49.1% stevioside. This continuous, sustainable process enhances purity and scalability for industrial applications.

## Data Availability

The data presented in this study are available on request from the corresponding author.
